# *In Vitro* Nanobody Library Construction by Using Gene Designated-Region Pan-Editing Technology

**DOI:** 10.34133/2022/9823578

**Published:** 2022-08-01

**Authors:** Zhiyuan Niu, Zhixia Luo, Pengyang Sun, Linwei Ning, Xinru Jin, Guanxu Chen, Changjiang Guo, Lingtong Zhi, Wei Chang, Wuling Zhu

**Affiliations:** ^1^Synthetic Biology Engineering Lab of Henan Province, School of Life Sciences and Technology, Xinxiang Medical University, Xinxiang, 453003 Henan, China; ^2^Department of Oncology, Xinxiang First People’s Hospital, The Affiliated People’s Hospital of Xinxiang Medical University, Xinxiang 453000China

## Abstract

Camelid single-domain antibody fragments (nanobodies) are an emerging force in therapeutic biopharmaceuticals and clinical diagnostic reagents in recent years. Nearly all nanobodies available to date have been obtained by animal immunization, a bottleneck restricting the large-scale application of nanobodies. In this study, we developed three kinds of gene designated-region pan-editing (GDP) technologies to introduce multiple mutations in complementarity-determining regions (CDRs) of nanobodies *in vitro*. Including the integration of G-quadruplex fragments in CDRs, which induces the spontaneous multiple mutations in CDRs; however, these mutant sequences are highly similar, resulting in a lack of sequences diversity in the CDRs. We also used CDR-targeting traditional gRNA-guided base-editors, which effectively diversify the CDRs. And most importantly, we developed the self-assembling gRNAs, which are generated by reprogrammed tracrRNA hijacking of endogenous mRNAs as crRNAs. Using base-editors guided by self-assembling gRNAs, we can realize the iteratively diversify the CDRs. And we believe the last GDP technology is highly promising in immunization-free nanobody library construction, and the full development of this novel nanobody discovery platform can realize the synthetic evolution of nanobodies *in vitro*.

## 1. Introduction

Nanobodies provide the remarkable specificity of antibodies within the 15 kDa single-variable domains (VHH) of the heavy-chain-only antibodies found in llamas and other camelids [1]. The advantages of nanobodies include their ability to bind with high affinity to epitopes that are inaccessible to traditional antibodies (≥150 kDa), better stability and the possibility of lower immunogenicity, and Lego-like modularity, and they even increase the efficacy of chimeric antigen receptor (CAR) T cells [2, 3]. There are dozens of active nanobody programs of all stripes in clinical development, covering lots of targets and indications [4–6], even in the diagnosis and treatment of COVID-19 [7, 8]. However, nanobodies discovered to date have been derived from the immunization of camelids [9], which is invariably time-consuming, expensive, and unreliable in quality. Therefore, in this study, we are dedicated to developing a novel platform technology that can be used to expedite the development of nanobodies for therapeutic and diagnostic applications by avoiding the need for animal immunization.

There are mainly two kinds of previously described strategies for *in vitro* library creation or introducing variation in antibody complementarity-determining region (CDR) loops: the static antibody libraries and the dynamic antibody libraries. The static antibody libraries’ sequence diversity remains constant after it is generated. This library construction scheme mainly relies on the artificial synthesis of antibody sequences and the artificial introduction of sequence diversity in the CDRs of antibodies. For example, libraries that are generated by amplifying CDR sequences from natural antibodies before grafting them into recombinant display systems for selection *in vitro* [10], using trinucleotide assembly of mutagenic oligonucleotides to create naive libraries on a single antibody framework (i.e., antibody constant region) or a collection of antibody frameworks [11–13], using synthetic libraries in which the antigen-binding sites were generated by diversifying only four kinds of amino acids (Tyr, Ser, Ala, and Asp) in CDRs [14], and generating synthetic antibody libraries, whose CDRs contain only two kinds of amino acids, including tyrosine and serine [15, 16]. By using these methods, we can accurately limit mutations to specified regions, like CDRs, or design positional amino acid frequencies in CDRs. However, this library building scheme strongly relies on synthetic antibody sequences and artificially introduces sequence diversity in CDRs. Also, binders acquired from these libraries are inferior in quantity and affinity to those obtained from the natural immune system. The dynamic antibody libraries’ antibody diversity is continuously generated. For example, use the most mutagenic RNA viral class, *Alphavirus sindbis* viruses as vectors for heredity and diversity, which achieved 24-hour selection cycles surpassing 10^-3^ mutations/base [17]. Libraries can also be constructed by coexpressing IgG, for example, the heavy chain (HC), light chain (LC), and activation-induced cytidine deaminase (AID) in mammalian cells. The expression of AID alone is sufficient to reproduce the salient features of somatic hypermutation (SHM) in both B cells and other mammalian cells [18, 19]. These currently available dynamic library construction methods allow an iterative fashion to diversify, display, and screen antibodies and more importantly, antibody affinity maturation via iterative cycles of antigen-coated magnetic beads sorting or fluorescence-activated cell sorting (FACS) under increasingly stringent sort conditions. This dynamic library allows for iteratively diversifying the antibody sequences; however, with a relatively low mutation efficiency, the mutations generated by RNA viruses and AID can hardly restrict to a small window of nucleotides within CDRs, and constant region mutations may cause antibody disfunction.

In summary, it is necessary to further develop a powerful *in vitro* library construction platform that combines the advantages of both precise mutations of CDRs in currently available static antibody libraries and iterative library building characteristics of dynamic antibody libraries. And this platform mimics the fine natural immune system and continuously generates diverse nanobodies for later displaying, screening, and maturation.

## 2. Materials and Methods

### 2.1. Reagents

Puromycin (P8230), Trytone (T8490), Agar Powder (A8190), Yeast Extract Powder (01-012), DNA purification kit (D1300), NaCl (S8210), Trypsin 0.25% (T1360), and PBS (D1040) were from Solarbio, Beijing, China. Stbl3 Chemically Competent Cells (KTSM110L) were from AlpaLife, Shenzhen, China. Polybrene (40804ES76) and Hieff Canace® High-Fidelity DNA Polymerase (10148ES10) were from Yeasen Biotech, Shanghai, China. The Tissue gDNA Isolation Kit (BW-GD2211) was from BIOMIGA, Zhe Jiang, China. EcoR I (1040S), Xba I (1093A), and T4 ligase (2011A) were form TAKARA, Da Lian, China. SanPrep Column DNA Gel Extraction Kit (B518131) and Pfu DNA Polymerase were purchased from Sangon Biotech, Shanghai, China. The E.Z.N.A.® Plasmid Midi Kit I (D6926-03) was purchased from Omega Bio-Tek, Guangzhou, China. Green Taq Mix (P131-01) was from Vazyme Biotech Co., Ltd, Nanjing, China. Goat anti-llama IgG H&L (FITC) (ab112785) was from Abcam, Shanghai, China. The Calcium Phosphate Cell Transfection Kit (C0508) was from Beyotime, Haimen, PR China.

### 2.2. Cell Culture

Dulbecco’s modified Eagle’s medium (DMEM) and fetal bovine serum (FBS) were from Gibco, Thermo Fisher, Shanghai, China. The HEK293T (GDC0187) cell line was obtained from China Center for Type Culture Collection (CCTCC, Wuhan, China) and cultured according to their instructions. All media were supplemented with 1% penicillin-streptomycin (C0222, Beyotime, Haimen, China) and 10% FBS. All experiments were performed with mycoplasma-free cells.

### 2.3. Plasmids

pCMV-BE3 (Addgene plasmid # 73021) was a gift from David Liu. Genes expressing LaG-2, LaG-2/G4, LaG-2/G4.1, LaG-2/G4.2, LaG-2/G4.3, AIDmut1, AIDmut2, and membrane-expressed LaG-2 (mLaG-2) were directly synthesized (GenScript, Nanjing, China) and subcloned into pCDH-CMV-MCS-EF-1*α*-Puro. Genes expressing 3×gRNA, 3×gRNA*Δ*, 3×gRNA*Δ*15, and 3×gRNA*Δ*21 were directly synthesized (GenScript, Nanjing, China) and subcloned into pLL7-U6-MCS-EF-1*α*-Blast. The full sequences of these genes or plasmids were provided in Table S1.

### 2.4. Lentivirus Packaging and Infection

Transfection of HEK293T was performed by using Calcium Phosphate Cell Transfection Kit according to the manufacturer’s instructions. We use the vector for expressing LaG-2/G4 (pCDH-CMV-LaG-2/G4-EF-1*α*-Puro), LaG-2/G4.1, LaG-2/G4.2, LaG-2/G4.3, or LaG-2 (pCDH-CMV- LaG-2-EF-1*α*-Puro) : viral packaging vector (psPAX2) : viral envelope vector (pMD2G) at 4 : 3 : 1 ratio. Lentivirus containing supernatant was harvested at both 48 and 72 h after transfection and was filtered using a 0.45 *μ*m filter. The acquired lentivirus was mixed with the PEG solution (5% PEG8000 and 0.5 M NaCl) overnight and concentrated by centrifugation at 3000 g for 30 min at 4°C.

Dilution of lentivirus in DMEM complete +10 *μ*g ml^-1^ polybrene was used for HEK293T infection. HEK293T-expressing LaG-2/G4, LaG-2/G4.1, LaG-2/G4.2, LaG-2/G4.3, or LaG-2 were initially selected on 5 *μ*g ml^-1^ puromycin and subsequently grown in the presence of 1 *μ*g ml^-1^ puromycin.

### 2.5. HEK239T Transfection

Transfection of HEK293T was performed by using the Calcium Phosphate Cell Transfection Kit according to the manufacturer’s instructions. $1\times 10^{6}$ HEK293T cells expressing LaG-2 were seeded in 6-well plates. At 1 d, 3 d, and 5 d postseeding, cells were transfected 3 times with 4.5 *μ*g plasmids expressing BE3, AIDmut1, or AIDmut2 base-editors, and 1.5 *μ*g plasmids expressing 3×gRNA, 3×gRNA*Δ*, 3×gRNA*Δ*15, or 3×gRNA*Δ*21.

### 2.6. Genomic DNA Extraction and Polymerase Chain Reaction

Genomic DNA was isolated from HEK293T cells 48 h after the third time of transfection by using the Tissue gDNA Isolation Kit according to the manufacturer’s instructions. Three nested pairs of primers were designed for LaG-2/G4, LaG-2/G4.1, LaG-2/G4.2, and LaG-2/G4.3 PCR amplification. The primer sequences used for nested PCR were as follows: Nest 1F: $5^{\prime }$-AGCTCGTTTAGTGAACCGTC-$3^{\prime }$, Nest 1R: $5^{\prime }$-CACAGCAGGCCGTTGAACTTG-$3^{\prime }$, Nest 2F: $5^{\prime }$-TCCACGCTGTTTTGACCTCC-$3^{\prime }$, Nest 2R: $5^{\prime }$-CACCTGTAGAAGGGGTTCTC-$3^{\prime }$, Nest 3F: $5^{\prime }$-ATATTCTAGATGGCCCTGCTGCTGCAC-$3^{\prime }$, and Nest 3R: $5^{\prime }$-ATATGAATTCCTCGCAGGTGCCCTGGTT-$3^{\prime }$. The primer combination was Nest 3F/Nest 3R for LaG-2 PCR amplification. The reaction conditions consisted of initial denaturation at 98°C for 3 min, 35 cycles of 98°C for 10 s, 68°C for 40 s, and a final extension step for 5 min at 72°C.

### 2.7. Flow Cytometry

Flow cytometry samples were run on a Guava easyCyte 8HT. The data were analyzed by using the FlowJo software (v. 7.6.4, TreeStar). The statistics presented are based on at least 10,000 events gated on the population of interest.

### 2.8. Experimental Repeats and Statistics

All assays had been performed at least thrice to ensure the repeatability of the experiments. The representative pictures and statistical analysis were shown in the final figures. Data for all experiments were analyzed with the Python applets we wrote.

## 3. Results

### 3.1. Nanobody Library Construction by Using G-Quadruplex Fragments

Recently, the Liu group developed a series of base-editors, which could benefit point mutation correction by efficiently converting one base pair to a different base pair, such as adenine base-editors (ABEs) [20] and cytosine base-editors (CBEs) [21] or benefit disruption of specific regions and multiplex base-editing applications, such as the ABE8e base-editor [22]. These base-editors will not induce double-stranded DNA breaks or extensive insertions and deletions (indels) to target sites, cause frameshift mutations, and yield a nonsense protein. These advantages make the base-editor an excellent choice for creating mutations in antibody CDRs. However, base-editors only operate on single-stranded DNA but reject double-stranded DNA. Still, this feature is critical to restrict deaminase activity to a small window of nucleotides within the single-stranded DNA structures.

G-quadruplex is a specialized DNA secondary structure consisting of a sequence of guanine-rich nucleic acids. The sequence forming a G-quadruplex requires at least 4 consecutive groups of G bases, joined by 1-7 arbitrary bases and not less than 2 G bases per group (G_(≥2)_N_1-7_G_(≥2)_N_1-7_G_(≥2)_N_1-7_G_(≥2)_) [23, 24]. The formation of the G-quadruplex allows its complementary strand to exist in a single-stranded state, so gene editing can be performed within the window by finding a protein that directs deaminase to the complementary single strand of the G-quadruplex.

**Figure 1:**
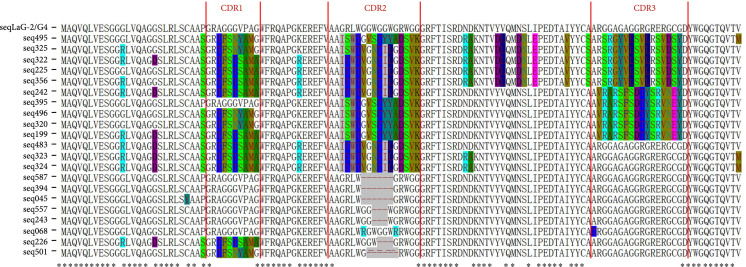
Sequences of LaG-2/G4 and spontaneously mutated LaG-2/G4. The CDRs were marked with red lines. Bases marked with colored backgrounds are the spontaneously mutated bases, and “-” marks the position where the base deletion mutation occurred.

Based on the above facts, the first nanobody library construction strategy we tried was starting from a consensus framework derived from an anti-GFP nanobody LaG-2 [25]. CDR1, CDR2, and CDR3 of nanobody LaG-2 were replaced by the natural G-quadruplex fragments from *VEGF*, *c-myc*, and *BCL-2* genes, respectively [26], modified LaG-2 was named LaG-2/G4, and the sequence was shown in Figure S1A. And base-editors were directed to the single-strand window by nucleolin, which promotes the formation of G-quadruplexes and stabilizes the structure of G-quadruplexes when it is bound to them. Therefore, it stands to reason that base-editors tethered to nucleolin could be directed to G-quadruplex fragments containing CDRs of LaG-2, as shown in schematic Figure S1B. However, we found that G-quadruplex sequences containing nanobodies could spontaneously generate mutations without the use of nucleolin-tethered base-editors when nanobody sequences were integrated into 293T cells genomes by lentiviral infection. The results showed that the mutations were mainly concentrated in three CDRs, with only a few mutations outside, the overall mutation rate was 161/1000 bp^-1^ (per bp) (Figure 1), and data of the mutation sites and types, together with the ratio of four nucleotides before and after mutation, were shown in Figure S2. Also, we noticed 3 or 6 amino acids deletions in CDR2; however, these deletions did not cause frameshift mutations. Unfortunately, these mutations lack randomness, and both the mutation sites and the mutated amino acids are preferential. These characteristics are detrimental to the library construction. Moreover, we proved that LaG-2/G4 is stable in the Stbl3 *E. coli* strain (Figure S3), and high-fidelity PCR is capable of introducing a small number of mutations but far fewer than those introduced by amplification of LaG-2/G4 in 293T cells (Figure S4). To further clarify the pattern of mutations induced by G-quadruplex fragments, we tried to switch CDR2 and CDR3 regions of LaG-2/G4 (Figure S5A) or replaced CDR2 containing G-quadruplex fragments with another G-quadruplex fragment from *HIF-1α* (Figure S5B) or *RET* gene (Figure S5C). Then these LaG-2/G4 variants were amplified by Hieff Canace® High-Fidelity DNA Polymerase, and mutations generated by PCR were shown. Moreover, CDR2 containing G-quadruplex fragments from *RET* genes were amplified by Pfu DNA Polymerase (Figure S5D). However, all these tries failed to improve the diversity of G-quadruplex-induced mutations. More importantly, G-quadruplexes are difficult to amplify in PCR reactions, so it is possible that there is mutant-preferred amplification, and the mutations generated in PCR reactions or HER293T cells are much less than they appeared. Moreover, error-prone PCR has the potential to increase G-quadruplex-induced mutations; however, this does not meet our initial purpose, which involved the G-quadruplex and base-editor. Also, error-prone PCR would accumulate mutations in the constant regions of the nanobody, which could potentially lead to the nanobody’ incorrect folding and loss of function. So we quit our plan to use G-quadruplex to induce diversity in the CDRs of nanobodies in this study.

### 3.2. Nanobody Library Construction by Using Traditional gRNA-Guided and nCas9-Tethered Base-Editors

**Figure 2:**
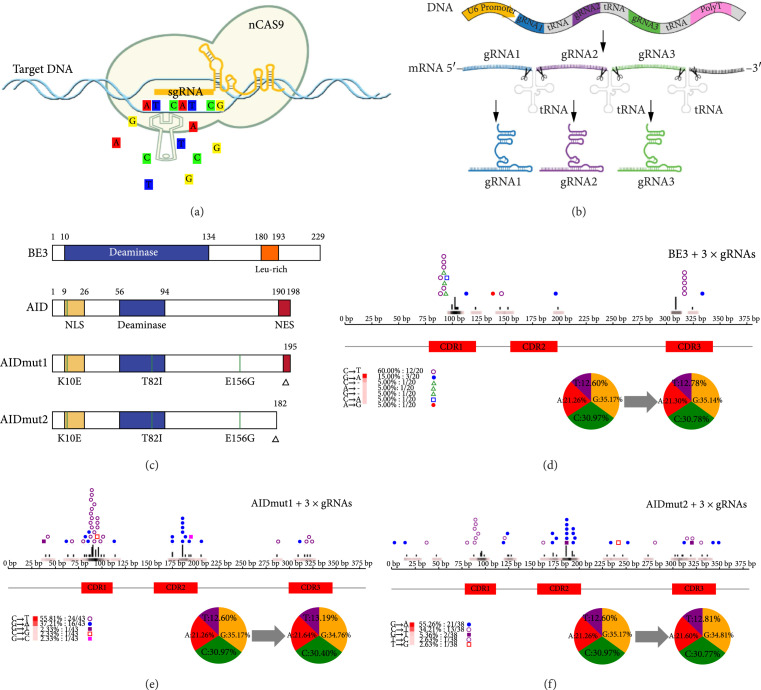
Gene designated-region pan-editing mediated by conventional gRNA-guided and nCas9-tethered base-editors. (a) Schematic of nCas9-tethered base-editor-mediated gene designated-region pan-editing (GDP). (b) Three gRNAs expressed by the polycistronic-tRNA-gRNA (PTG) system. (c) Diagram of BE3, AID, and AID variants. NES, deaminase domain, truncations, and activity altering mutations are indicated. (d–f) HEK293T cells containing LaG-2 were transfected with indicated combinations of (d) BE3, (e) AIDmut1, or (f) AIDmut2 and 3×gRNAs for 3 times; then the LaG-2 loci were sequenced. Graphs of the enrichment of mutation at each base are shown here; we also indicate the type and number of mutations in the lower-left corner, and “-” means deletion mutation. The pie chart represents the change in the ratio of the four bases before and after the mutation. In (d–f), mutations from at least 10 LaG-2 sequences were analyzed.

Besides G-quadruplex, Cas9/gRNA complexes are also perfect choices to create single-stranded bubbles on nanobody CDRs. So in this section, we try to use gRNAs to direct Cas9-tethered base-editors to nanobody CDRs, as shown in Figure 2(a). The gRNA/Cas9 base-editor complex is recruited to the target sequence by the base-pairing between the gRNA sequence and the complement to the target sequence in the genomic DNA, and then multiple mutations were generated. To create more mutations, we decide to diversify all three CDRs on nanobodies, and this requires at least three gRNAs. Here, we use a polycistronic-tRNA-gRNA (PTG) strategy [27] to quickly assemble three gRNAs in one construct, in which, three CDR-targeting gRNAs are flanked by glycine tRNAs to create polycistronic glycine tRNA-gRNA constructs. Taking advantage of the endogenous tRNA processing system in mammalian cells, we can efficiently transcript three CDR-targeting gRNAs driven by only one Pol III promoter (Figure 2(b)). Three different nCas9-tethered base-editors were evaluated for nanobody library construction (Figure 2(c)). The BE3 base-editor (APOBEC–XTEN–nCas9–UGI) was engineered in the lab of David R. Liu [21]: AIDmut1 base-editor (AID∗*Δ*–nCas9), in which, AID∗*Δ* is a mutated wild type AID (K10E + T82I + E156G) with partial depletion (196-198AA) of its C terminus nuclear exporting sequence (NES) which ablates its nuclear export signal while increasing somatic hypermutation (SHM). AID∗*Δ* was developed by Hess et al. [28], and we tethered this AID variant to nCas9, thus forming the AIDmut1 base-editor; the last base-editor is AIDmut2, which is generated by removing the full length of NES (183-198AA) from the AIDmut1 base-editor, and deletion of the full-length NES was proved to create more diversification on target site than partial deletion, according to Ma et al. [29].

To test the resulting base-editors, we transfected HEK293T cells which preintegrated the LaG-2 nanobody sequence in the genome, with two-plasmid mixtures in which one plasmid expresses three CDRs-targeting gRNAs and another expresses the base-editor. Transfection was conducted three times, to facilitate the accumulation of mutations. The results demonstrated that the overall mutation rates of BE3, AIDmut1, and AIDmut2 were 2.92/1000 bp^-1^, 10.2/1000 bp^-1^, and 6.23/1000 bp^-1^, respectively. AIDmut1 and AIDmut2 substantially increased the base-editing efficiency compared with BE3. SHM introduces nucleotide alterations at the V regions of heavy and light chain genes at a rate of 1/1000 bp^−1^ [30], which is enough to enable the selection of B cells producing high-affinity antibodies. So, the mutation rates of tested base-editors, especially AIDmut1 and AIDmut2, were far beyond SHM. The base-editing active window spans from −10 to −50 (counting the PAM as positions 1–3) at three CDRs loci (Figures 2(d)–2(f)). Moreover, BE3 and AIDmut1 caused mainly C-to-T conversion (60% and 55.81%, respectively), while AIDmut2 was mainly G-to-A mutation (55.26%). Meanwhile, low levels of unexpected A/T conversion or deletion were observed for BE3 and AIDmut2. Notably, rare long-range fragment deletion was observed for AIDmut1 and AIDmut2 (Figure S6); this is not likely the features of base-editors, so we excluded these minor sequences from analysis in Figures 2(e) and 2(f). And there are no significant differences between the ratio of four nucleotides before and after mutation for all of the base-editors (Figures 2(d)–2(f)). In the collection, we propose that expressing AIDmut1 and AIDmut2 base-editors were feasible strategies for nanobody library construction *in vitro*.

### 3.3. Nanobody Library Construction by Using Self-Assembling gRNA-Guided and nCas9-Tethered Base-Editors

Based on the above findings, we know that the expression of three gRNAs can effectively guide the base-editors to the three CDRs and cause mutations in the gRNA recognition region and adjacent sequences (Figure S7). However, gRNA-target recognition relies mainly on the 20 bp seed region at the $5^{\prime }$ end, and a mutation of only 2 bp in the seed region results in a 96% loss of gRNA guidance function [31]. Also, our data demonstrated that mutations generated by AID mutants pile up at a very slow rate. On average, there are only 4 mutation sites on each nanobody sequence, after three times of transfection and mutation (Figure S7). Therefore, when base-editors are guided by the conventional gRNAs, mutations that emerged from the first round of library construction will prevent the generation of more mutations in the next round.

**Figure 3:**
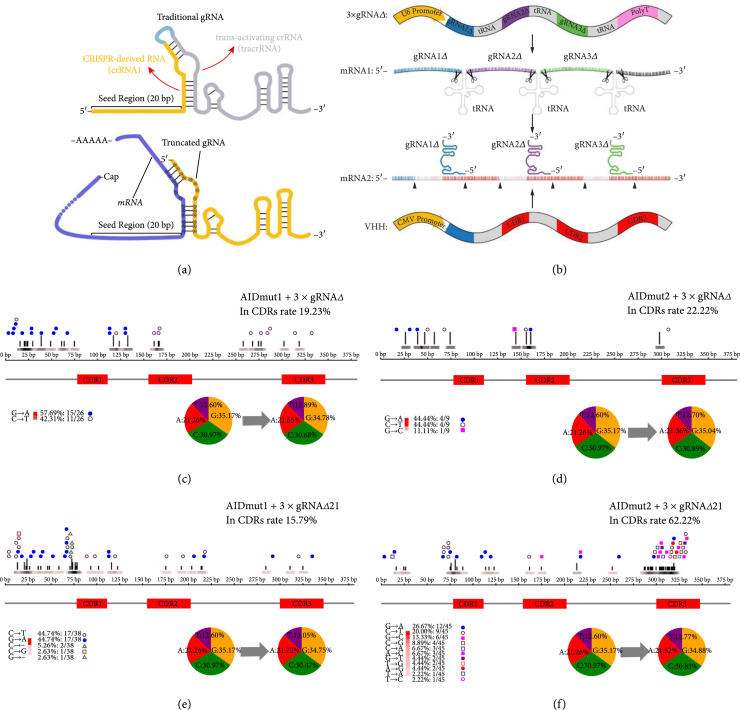
Gene designated-region pan-editing mediated by self-assembling gRNA-guided and nCas9-tethered base-editors. (a) Structures of conventional gRNA and truncated gRNA. (b) Schematic summary of the formation of three self-assembling gRNAs. (c–f) HEK293T cells containing LaG-2 were transfected with indicated combinations of (c) AIDmut1 and 3×gRNA*Δ*, (d) AIDmut2 and 3×gRNA*Δ*, (e) AIDmut1 and 3×gRNA*Δ*21, and (f) AIDmut2 and 3×gRNA*Δ*21 for 3 times; then the LaG-2 loci were sequenced. Graphs of the enrichment of mutation at each base are shown here; we also indicate the type and number of mutations in the lower-left corner, and “-” means deletion mutation. The pie chart represents the change in the ratio of the four bases before and after the mutation. In (c–f), mutations from at least 10 LaG-2 sequences were analyzed.

To iteratively diversify the CDRs, firstly, we reviewed the process of generation of conventional gRNA [32], which is a combination of the endogenous bacterial CRISPR RNA (crRNA) and transactivating crRNA (tracrRNA) into a single chimeric guide RNA (gRNA) transcript. The gRNA combines the targeting specificity of the crRNA with the scaffolding properties of the tracrRNA into a single transcript (Figure 3(a), upper panel). We speculate that the formation of the functional CRISPR/Cas9 complex has no mandatory requirement for the crRNA-tracrRNA-hybridized sequence. This part of the sequence only needs to satisfy the stem-loop structure formed between crRNA and tracrRNA in traditional gRNAs, and we can take advantage of this feature to construct the self-assembling gRNAs. We designed truncated gRNA (gRNA*Δ*) which does not contain the crRNA part of conventional gRNA. Also, the 14 bp $5^{\prime }$ end tracrRNA sequence that pairs with crRNA was modified, which now pairs with the $3^{\prime }$ constant region adjacent to the CDRs of nanobody mRNA (Figure 3(a), lower panel, and Figure 3(b)). After the pairing of gRNA*Δ* and the nanobody mRNA, endogenous mammalian RNases will cleave mRNA (generating truncated-mRNA, mRNA*Δ*) and assist maturation of the gRNA*Δ* : mRNA*Δ* duplex (self-assembling gRNA), just as RNase III cleaves pre-crRNA base-paired with transactivating crRNA (tracrRNA) in the presence of Cas9 [33, 34]. The mature self-assembling gRNAs then guide nCas9-tethered base-editors to create mutations on target DNA sites. Most importantly, the seed regions of our self-assembling gRNAs always come from the latest mutated mRNA, while maintaining self-targeting ability, enabling continuously diversifying the CDRs.

The base-editing efficiency of self-assembling gRNA-guided base-editors was tested under the same condition when using conventional gRNAs. The results demonstrated that the overall mutation rates of AIDmut1 and AIDmut2 were 6.82/1000 bp^−1^ (Figure 3(c)) and 2.36/1000 bp^−1^ (Figure 3D), respectively. Both substantially decreased the base-editing efficiency compared with when they were guided by the conventional gRNAs. And more importantly, most of the mutations generated by AIDmut1 and AIDmut2 are outside the CDRs, which is unfavorable for an antibody library building. We speculate that the self-assembling gRNAs may not be processed smoothly and produced three independently functioning self-assembling gRNAs. Also, excess mRNA sequences have the potential to cause single-strand range expansion, which in turn causes an expanded editing window.

As we mentioned above, RNase III cleaves pre-crRNA base-paired with tracrRNA in the presence of Cas9. Although we do not know which endogenous mammalian RNases cleave gRNA*Δ* : mRNA duplex, we speculate that mammalian RNases working as RNase III cleave the double-stranded RNA after the formation of gRNA*Δ* : mRNA : Cas9 trimer. It stands to reason that prolonging the $5^{\prime }$ end of gRNA*Δ* could facilitate RNases binding and cleavage of mRNA (Figure S8A). So we tested the base-editing ability of AIDmut1 and AIDmut2 guided by $5^{\prime }$ end prolonged 21 bp gRNA*Δ* (gRNA*Δ*21). The results demonstrated that the overall mutation rates of AIDmut1 and AIDmut2 were 9.97/1000 bp^−1^ (Figure 3(e)) and 10.7/1000 bp^−1^ (Figure 3(f)), respectively, both substantially increased the base-editing efficiency compared with when they were guided by the shorter gRNA*Δ*. Moreover, mutations are more concentrated in CDRs for AIDmut2 guided by gRNA*Δ*21, 62.22% of mutations are in CDRs (Figure 3(f)). Since nanobody mRNAs were engaged in generating self-assembling gRNAs, to test whether the loss of mRNAs would affect the subsequent expression and screening of nanobodies in the future, we display nanobodies on the surface of 293T cells by the addition of an N-terminal signal peptide and a C-terminal transmembrane domain. 293T cells containing membrane-expressed LaG-2 (mLaG-2) were transiently electroporated with AIDmut1 or AIDmut2 and self-assembling gRNAs. Then mLaG-2 expression was evaluated by flow cytometry, and we did not observe any loss in nanobody expression (Figures S8B and S8C). We speculate that this is because the U6 promoter is a weak promoter and only a small amount of gRNA*Δ*21 will be expressed, then hijack only a few endogenous mRNAs, and thus do not have a significant impact on the expression of nanobodies. We also observed the production of a small number of stop codons (Figures S8D and S8E), which again have not impaired the expression of the nanobodies as shown in Figures S8B and S8C.

**Figure 4:**
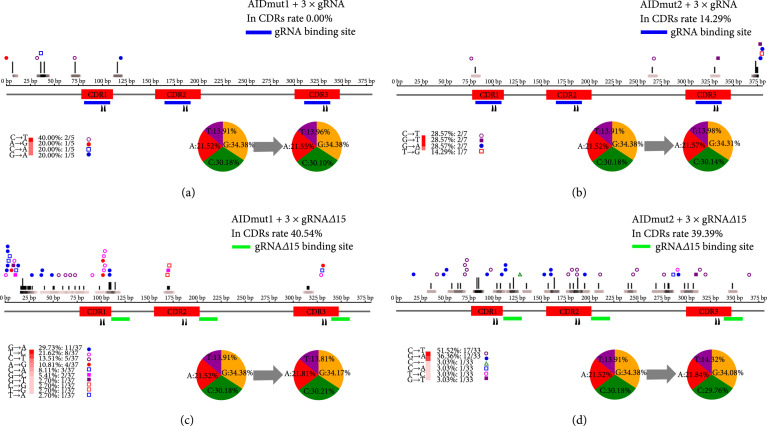
Comparison of mutation tolerance for base-editors guided by conventional gRNAs and self-assembling gRNAs. (a–d) HEK293T cells containing LaG-2 which contains 2 mutated bases on the 20 bp seed region recognition site (mutation sites marked with two black triangles) were transfected with indicated combinations of (a) AIDmut1 and 3×gRNA, (b) AIDmut2 and 3×gRNA, (c) AIDmut1 and 3×gRNA*Δ*15, and (d) AIDmut2 and 3×gRNA*Δ*15 for 3 times; then the LaG-2 loci were sequenced. Graphs of the enrichment of mutation at each base are shown here; we also indicate the type and number of mutations in the lower-left corner, and “-” means deletion mutation. The pie chart represents the change in the ratio of the four bases before and after the mutation. In (a–d), mutations from at least 10 LaG-2 sequences were analyzed.

To further confirm the iterative evolutionary capacity of our self-assembling gRNAs, we mutated 2 bases on the 20 bp seed region recognition site. Then, we tested the base-editing ability of AIDmut1 and AIDmut2 guided by $5^{\prime }$ end prolonged 15 bp gRNA*Δ* (gRNA*Δ*15). As expected, mutation of the seed region recognition site will not affect the binding of truncated gRNAs. Base-editors directed by self-assembling gRNAs substantially increased the base-editing efficiency compared with base-editors directed by conventional gRNAs; moreover, mutations are more concentrated in CDRs for base-editors guided by truncated gRNAs (Figures 4(a)–4(d)). The results demonstrated that the overall mutation rates of AIDmut1 and AIDmut2 directed by conventional gRNAs were 1.31/1000 bp^−1^ (Figure 4(a)) and 1.67/1000 bp^−1^ (Figure 4(b)), and the CDR rates were 0.00% and 14.29%, respectively. The overall mutation rates of AIDmut1 and AIDmut2 directed by gRNA*Δ*15 were 9.71/1000 bp^−1^ (Figure 4(c)) and 7.87/1000 bp^−1^ (Figure 4(d)), and the CDR rates were 40.54% and 39.39%, respectively. Thus, by using self-assembling gRNAs, we can realize the continuous diversification of nanobody CDRs.

## 4. Discussion

The emerging and developing of modern biotechnologies, such as synthetic biology, single base-editing, and photosensitive bioswitch, raise the feasibility of artificial evolution of molecules, cells, and even organisms. To realize the controlled evolution of nanobodies *in vitro*, we developed several gene designated-region pan-editing (GDP) technologies for library construction. We found that the self-assembling gRNA-guided base-editor is the most promising way to iteratively diversify the CDRs and construct the fully synthetic nanobody library. In each round of mutation, the effective library size was maintained at >10^7^. In the future, nanobody libraries acquired from a consensus framework derived from a humanized nanobody will greatly improve the safety of clinical applications of nanobodies.

However, more improvements are needed to totally abandon animal immunization and switch to *in vitro* techniques for producing nanobodies. For example, we still need to improve the efficiency and accuracy of self-assembling gRNA-guided base-editors. Moreover, we only tested CBEs in this study, to build more diverse nanobody libraries, and we also need efficient ABEs, but current ABEs are designed to correct for point mutations and thus cannot induce a wide range of mutations in specified regions of the gene. Therefore, we still need to redevelop the ABEs according to the requirements of library construction.

In this study, we observed theoretically and practically the potential of self-assembling gRNAs to consistently introduce random sequences in the gene designated-region. This has important implications not only for the preparation of nanobodies but also for the future introduction of artificial intelligence concepts into synthetic biology. For example, this technology allows the introduction of random function in cells, where we can randomly knock out or mutate any gene on the genome by reutilization of these random sequences with other Cas family members and other self-assembling gRNAs based on these random sequences. Then, by integrating existing photosensitive bioswitches and logic gates for signals input and output, we can design intelligent cells and obtain the desired protein molecules or cellular functions.

## Data Availability

DNA sequence data is supplied within the paper and in the supplementary materials.

## Supplementary Materials

Supplementary Materials Figure S1: G-quadruplex and nucleolin-tethered base-editor-mediated GDP technology. Figure S2: enrichment of the LaG-2/G4 spontaneous mutation at each base in HEK293T cells. Figure S3: enrichment of the LaG-2/G4 spontaneous mutation at each base in Stbl3. Figure S4: mutations on LaG-2/G4 generated by Hieff Canace® High-Fidelity DNA Polymerase. Figure S5: mutations on LaG-2/G4 variants generated by different High-Fidelity DNA Polymerases. Figure S6: amino acid mutations on LaG-2 that were generated by conventional gRNA-guided AIDmut1 and AIDmut2. Figure S7: mutations on LaG-2 DNA generated by conventional gRNA-guided AIDmut1. Figure S8: the characteristics of 3×gRNA*Δ*21-guided base-editors. Table S1: the full sequences of genes, plasmids, and mLaG-2.
