# Supplementary material for: In Vitro Nanobody Library Construction by Using Gene Designated-Region Pan-Editing Technology
Source: Biodes Res. 2022 Aug 1;2022:9823578. doi: 10.34133/2022/9823578 (PMC10521727; doi:10.34133/2022/9823578)
Supplement: Supplementary Materials — Figure S1: G-quadruplex and nucleolin-tethered base-editor-mediated GDP technology. Figure S2: enrichment of the LaG-2/G4 spontaneous mutation at each base in HEK293T cells. Figure S3: enrichment of the LaG-2/G4 spontaneous mutation at each base in Stbl3. Figure S4: mutations on LaG-2/G4 generated by Hieff Canace® High-Fidelity DNA Polymerase. Figure S5: mutations on LaG-2/G4 variants generated by different High-Fidelity DNA Polymerases. Figure S6: amino acid mutations on LaG-2 that were generated by conventional gRNA-guided AIDmut1 and AIDmut2. Figure S7: mutations on LaG-2 DNA generated by conventional gRNA-guided AIDmut1. Figure S8: the characteristics of 3×gRNAΔ21-guided base-editors. Table S1: the full sequences of genes, plasmids, and mLaG-2. [file 9823578.f1.zip › Supplementary Figure S1 - S8.docx]

**Supplementary Materials**


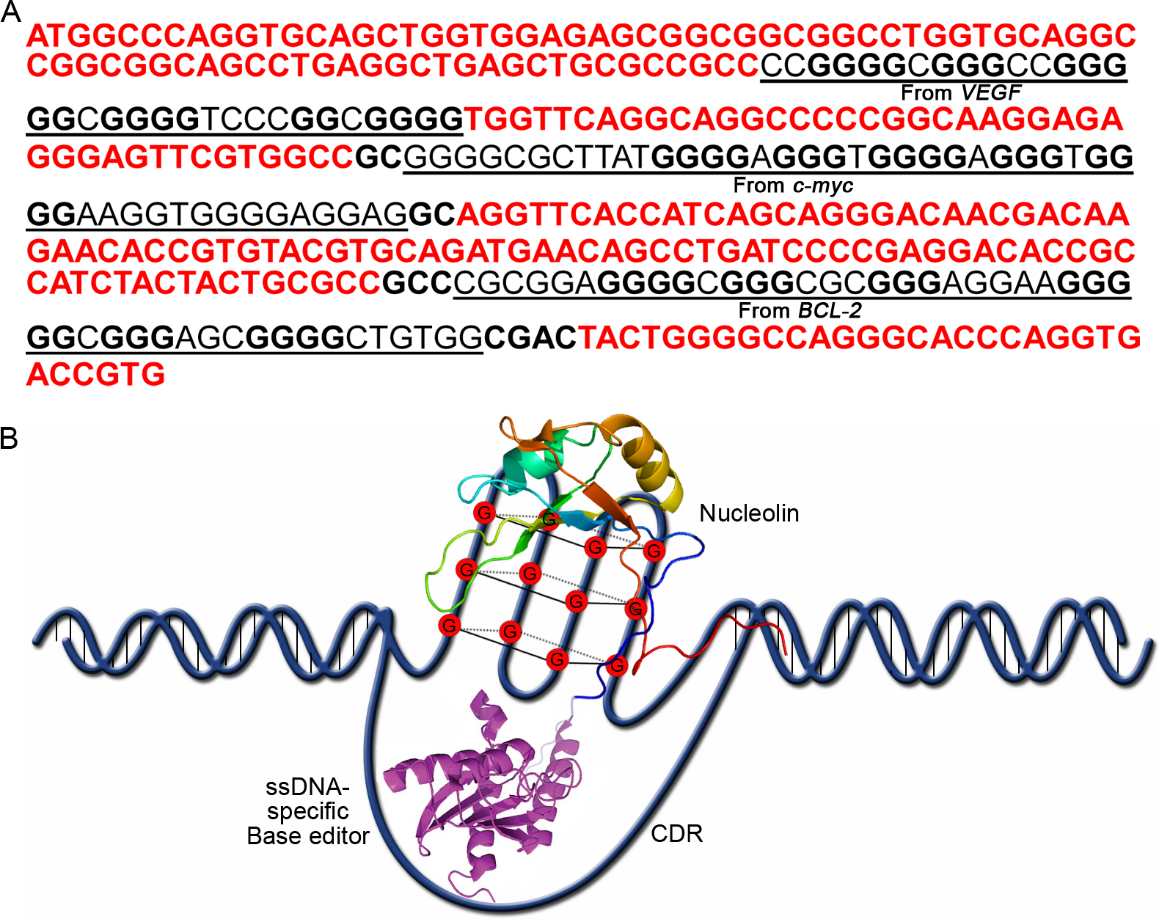


**Figure S1. G-quadruplex and nucleolin tethered base editor mediated GDP technology**

(A) The sequence of LaG-2/G4. (B) Schematic of nucleolin tethered base editor mediated gene designated-region pan-editing (GDP).

**
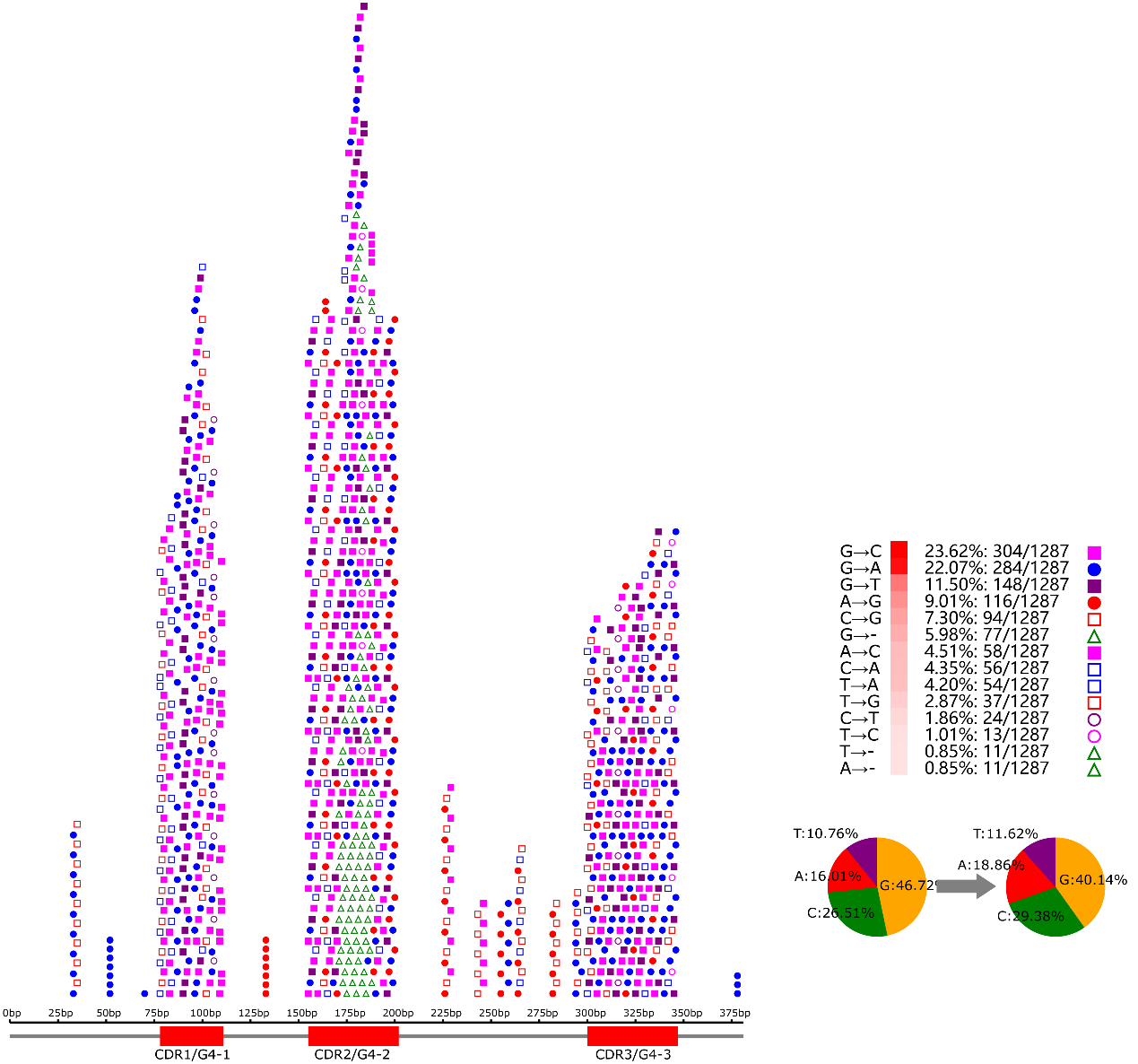
**

**Figure S2. Enrichment of the LaG-2/G4 spontaneous mutation at each base in HEK293T cells**

Figure S2 is related to Figure 1. LaG-2/G4 loci in HEK293T cells were subcloned to pCDH vector and transformed into Stbl3 Competent Cell, then LaG-2/G4 loci were sequenced. Graphs of the enrichment of mutation at each base are shown here, we also indicate the type and number of mutations in the lower-left corner, “-” means deletion mutation. The pie chart represents the change in the ratio of the four bases before and after the mutation. Mutations from 21 LaG-2/G4 sequences were analyzed.

**
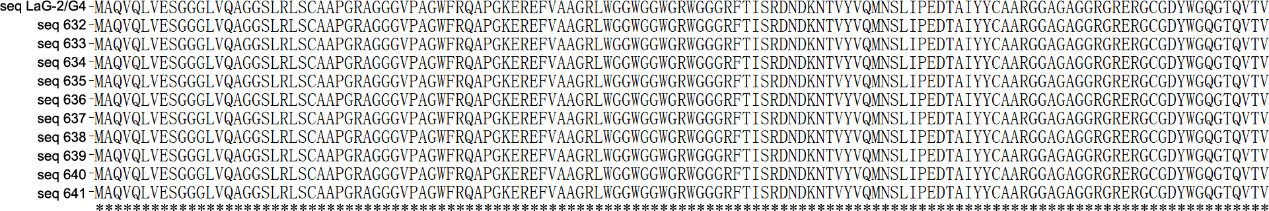
**

**Figure S3. Enrichment of the LaG-2/G4 spontaneous mutation at each base in Stbl3**

pCDH vectors containing LaG-2/G4 were directly transformed into Stbl3 Competent Cell, then LaG-2/G4 loci were sequenced.


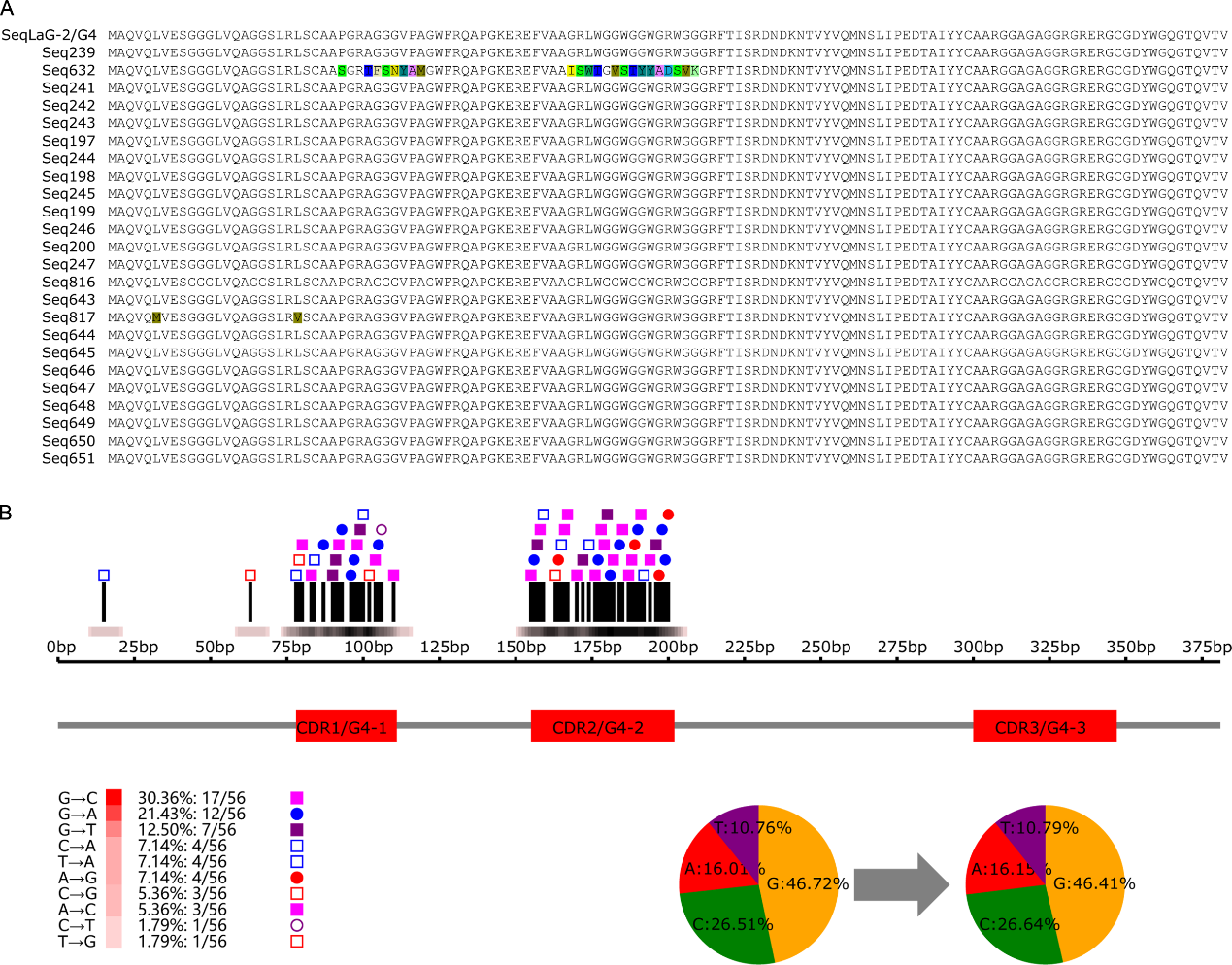


**Figure S4. Mutations on LaG-2/G4 generated by Hieff Canace® High-Fidelity DNA Polymerase**

Firstly, LaG-2/G4 sequences were amplified by Hieff Canace® High-Fidelity DNA Polymerase, and subcloned to pCDH vectors, then transformed into Stbl3 Competent Cell, and LaG-2/G4 loci were sequenced.

**
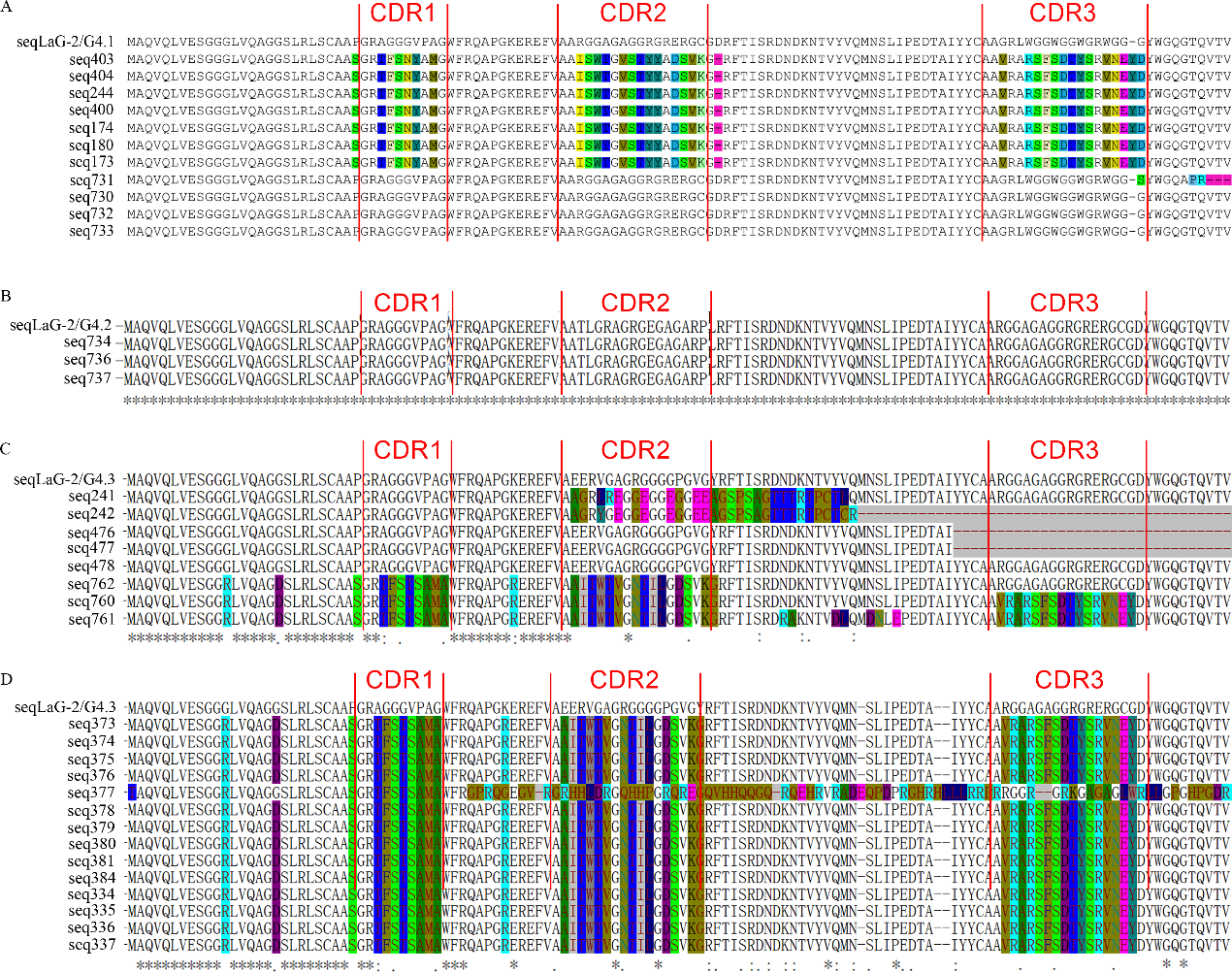
**

**Figure S5.** **Mutations on LaG-2/G4 variants generated by different High-Fidelity DNA Polymerases**

(A-D) Firstly, LaG-2/G4 variants were amplified by Hieff Canace® High-Fidelity DNA Polymerase (A-C) or Pfu DNA Polymerase (D), and subcloned to pCDH vectors, then transformed into Stbl3 Competent Cell, and LaG-2/G4 loci were sequenced. The first “-” in (A) and (C) means a stop codon was generated.

**
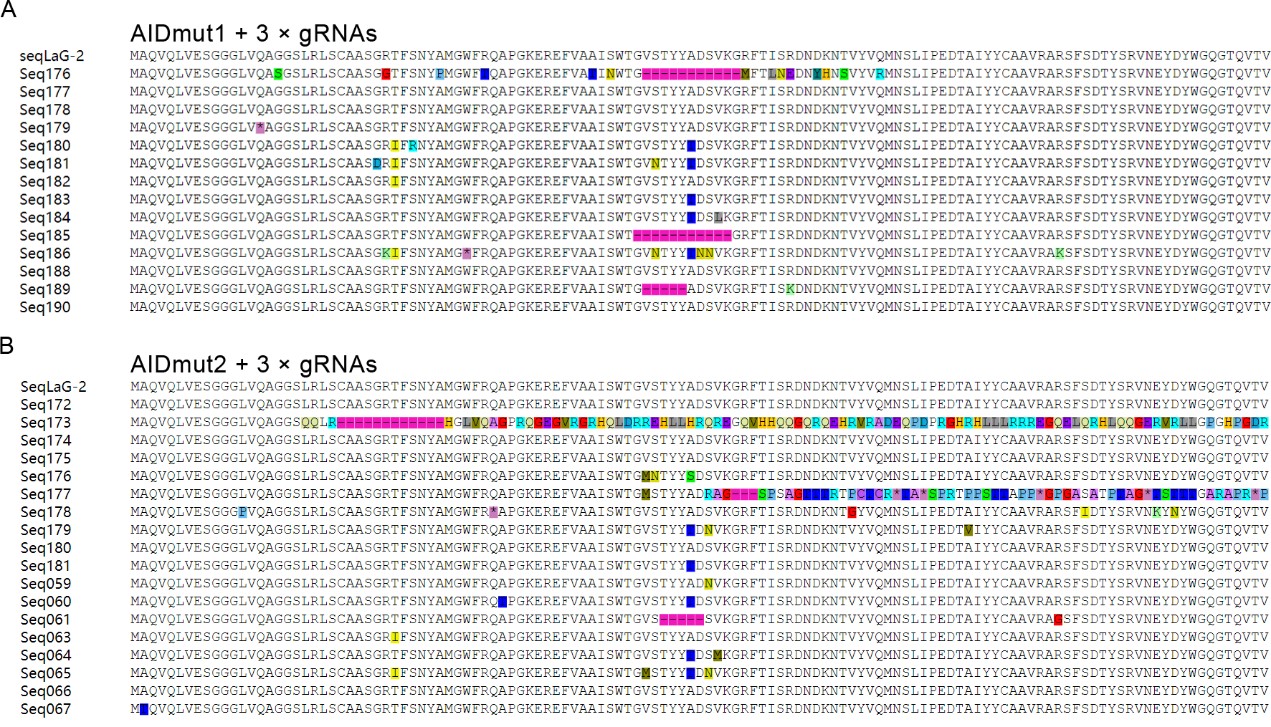
**

**Figure S6.** **Amino acid mutations on LaG-2 that were generated by conventional gRNAs guided AIDmut1 and AIDmut2**

Figure S6 is related to Figures 2E and 2F. Figure S6A is the translated amino acid sequence from the DNA sequence of Figure 2E, Figure S6B is the translated amino acid sequence from the DNA sequence of Figure 2F. Amino acids marked with colored backgrounds are the mutated amino acids, “-” means deletion mutation.

**
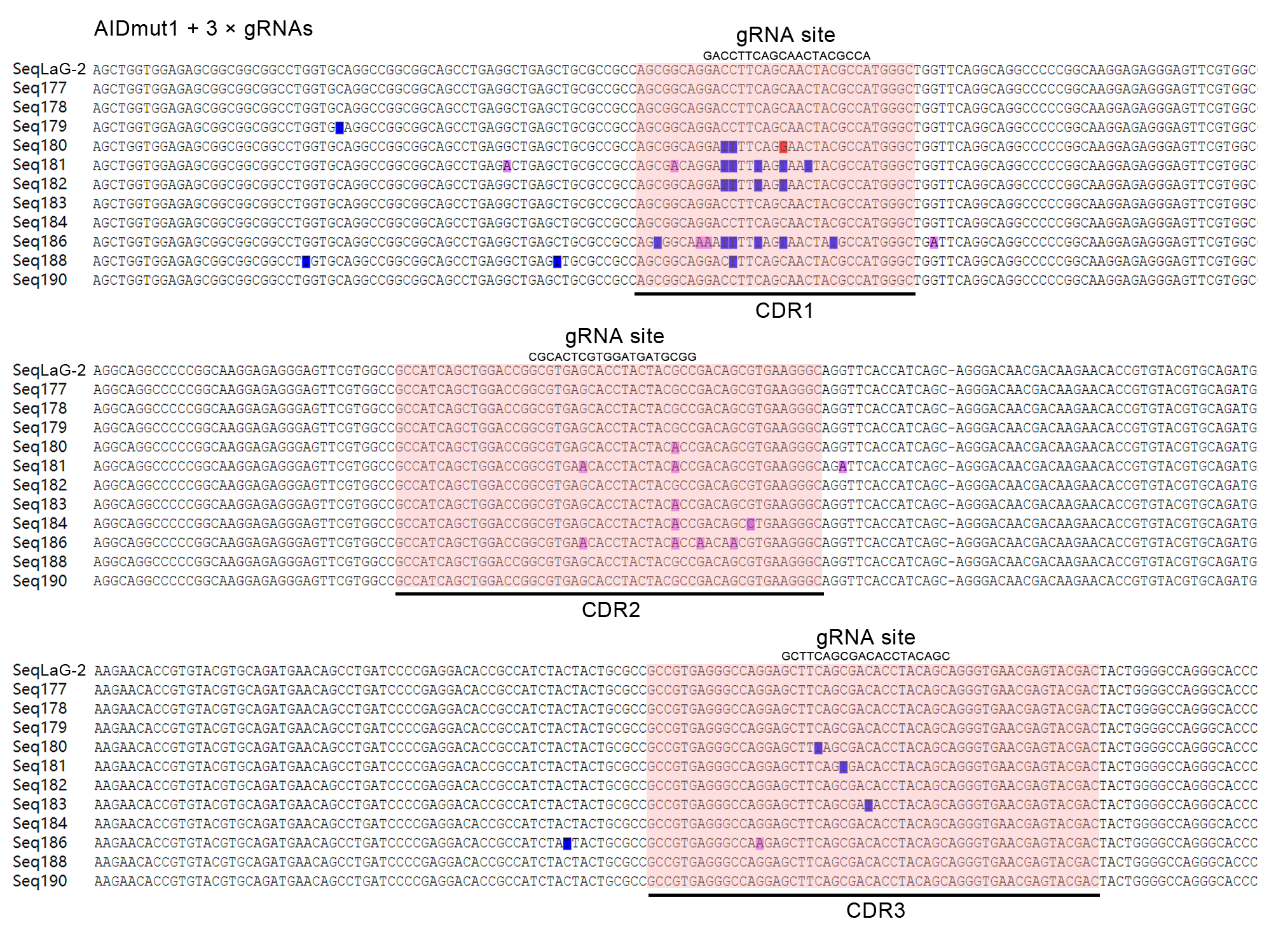
**

**Figure S7.** **Mutations on LaG-2 DNA generated by conventional gRNAs guided AIDmut1**

Figure S7 shows the DNA sequence of Figure 2E, bases marked with colored backgrounds are the mutated bases.

**
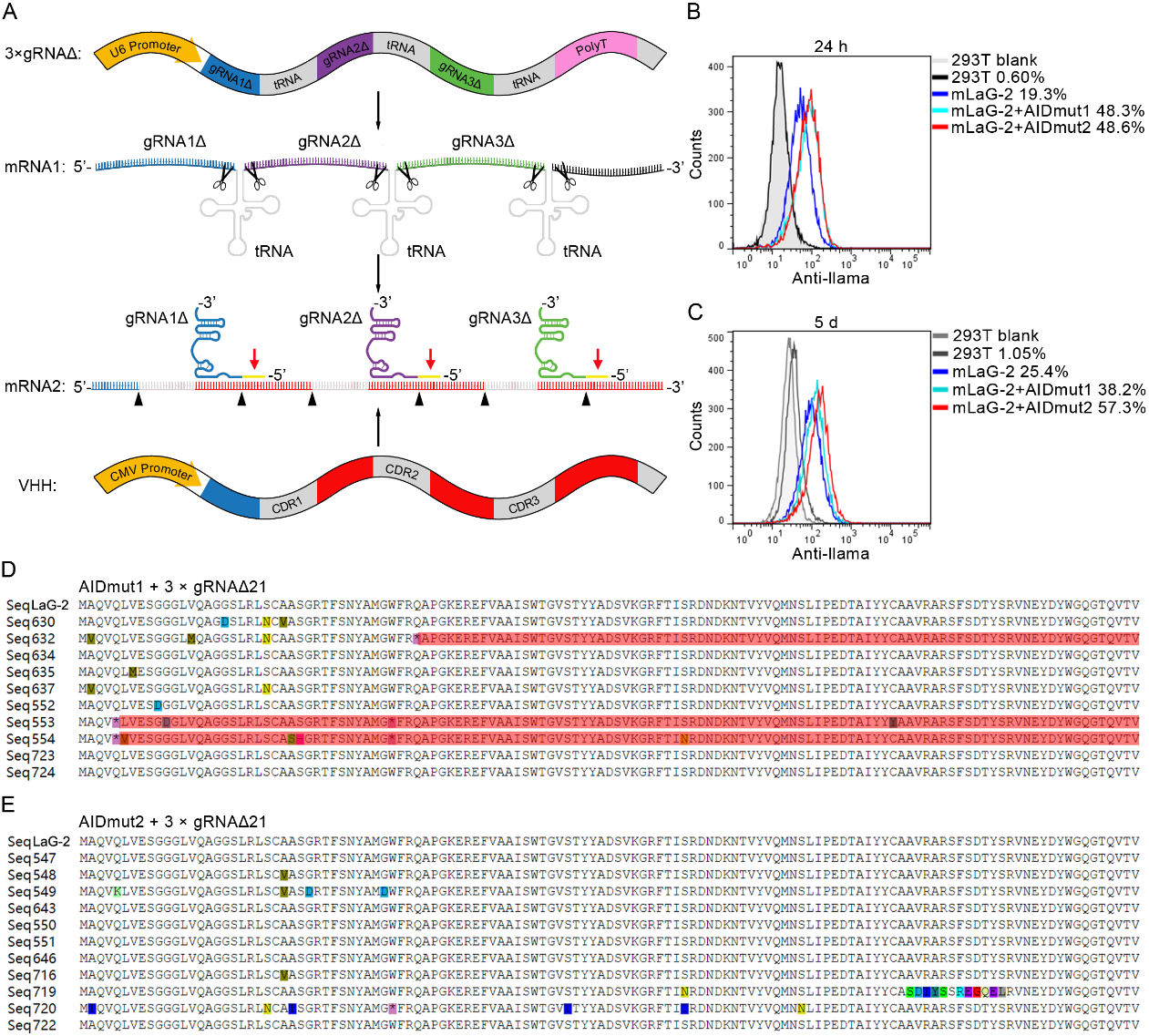
**

**Figure S8. The characteristics of 3×gRNAΔ21 guided base editors**

(A) Schematic of the 5′- end prolonged gRNAΔ. (B, C) HEK293T cells containing mLaG-2 were transiently electroporated with AIDmut1 and 3×gRNAΔ21 or AIDmut2 and 3×gRNAΔ21 for 3 times, then 24 h later (B) or 5 d later (C), the nanobody expression was evaluated by flow cytometry with goat anti-llama IgG H&L (FITC), which recognizes mLaG-2. 293T cells without mLaG-2 were incubated with PBS or goat anti-llama IgG H&L (FITC) and used as a blank or negative control. (D, E) Figure S8D is the translated amino acid sequence from the DNA sequence of Figure 3E, Figure S8E is the translated amino acid sequence from the DNA sequence of Figure 3F. Amino acids marked with colored backgrounds are the mutated amino acids, “*” represents a stop codon.
