# Supplementary figures and images for: In Vitro Nanobody Library Construction by Using Gene Designated-Region Pan-Editing Technology
Source: Biodes Res. 2022 Aug 1;2022:9823578. doi: 10.34133/2022/9823578 (PMC10521727; doi:10.34133/2022/9823578)

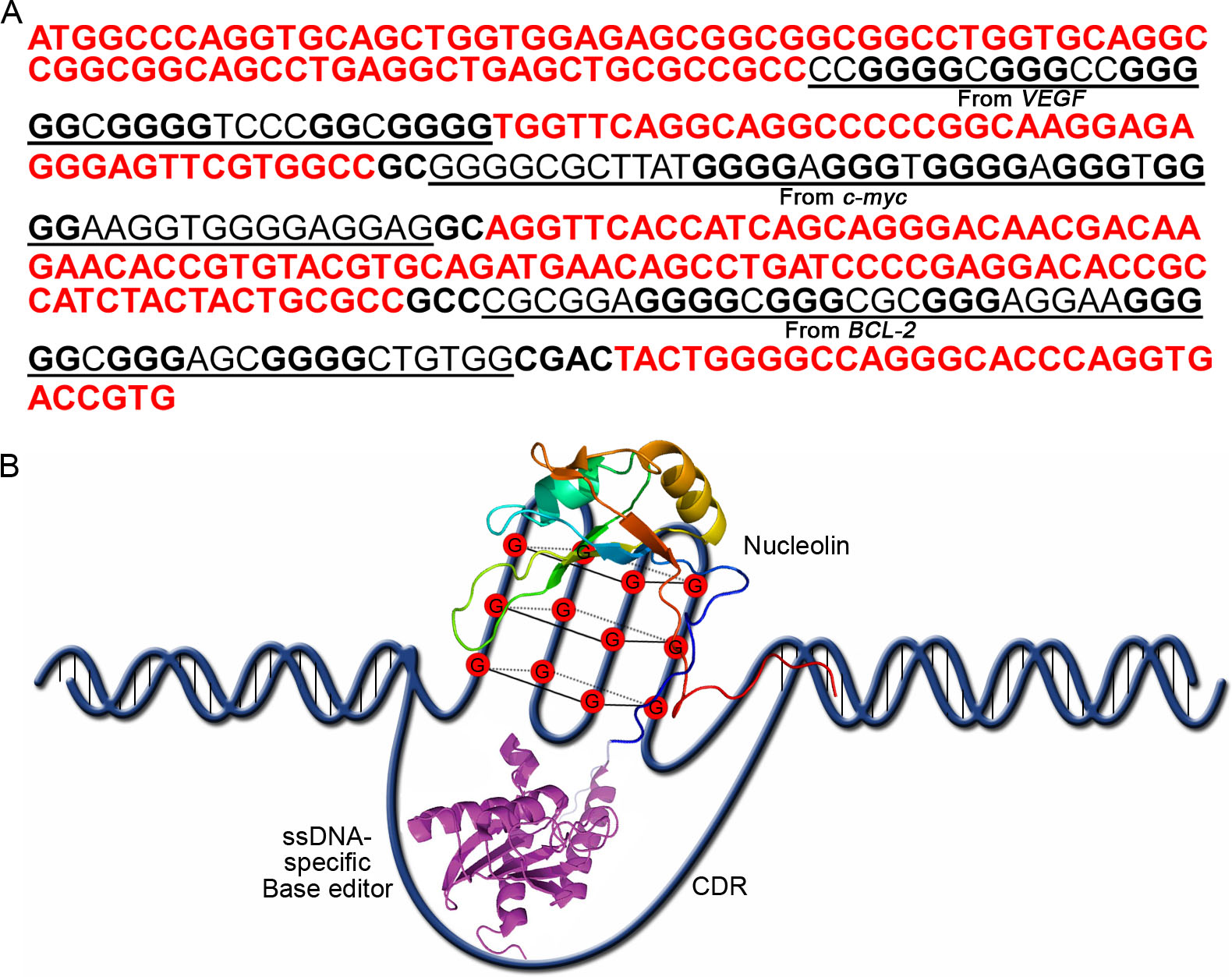

Supplement: Supplementary Materials — Figure S1: G-quadruplex and nucleolin-tethered base-editor-mediated GDP technology. Figure S2: enrichment of the LaG-2/G4 spontaneous mutation at each base in HEK293T cells. Figure S3: enrichment of the LaG-2/G4 spontaneous mutation at each base in Stbl3. Figure S4: mutations on LaG-2/G4 generated by Hieff Canace® High-Fidelity DNA Polymerase. Figure S5: mutations on LaG-2/G4 variants generated by different High-Fidelity DNA Polymerases. Figure S6: amino acid mutations on LaG-2 that were generated by conventional gRNA-guided AIDmut1 and AIDmut2. Figure S7: mutations on LaG-2 DNA generated by conventional gRNA-guided AIDmut1. Figure S8: the characteristics of 3×gRNAΔ21-guided base-editors. Table S1: the full sequences of genes, plasmids, and mLaG-2. [file 9823578.f1.zip › Figure S1.jpg]

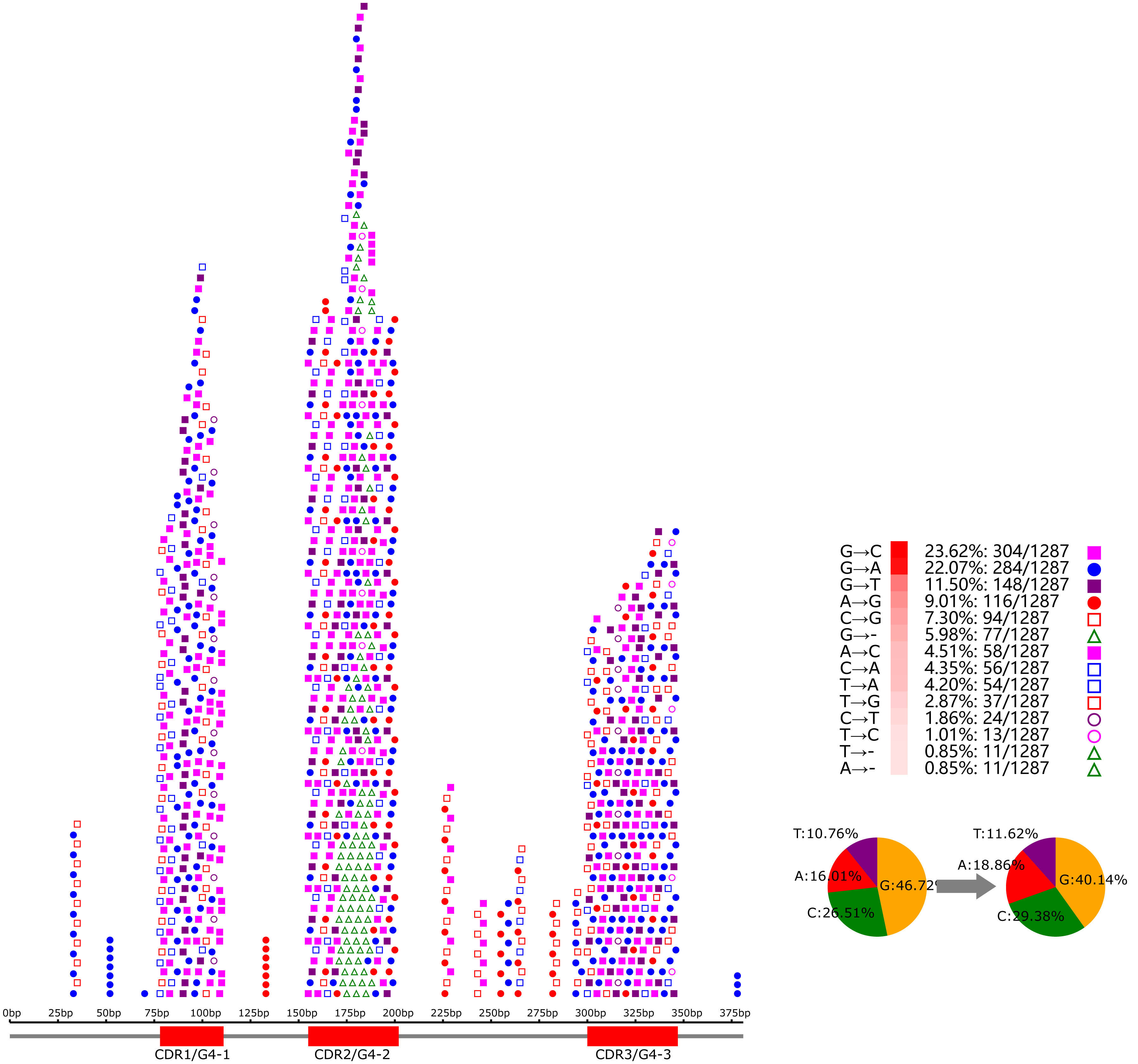

Supplement: Supplementary Materials — Figure S1: G-quadruplex and nucleolin-tethered base-editor-mediated GDP technology. Figure S2: enrichment of the LaG-2/G4 spontaneous mutation at each base in HEK293T cells. Figure S3: enrichment of the LaG-2/G4 spontaneous mutation at each base in Stbl3. Figure S4: mutations on LaG-2/G4 generated by Hieff Canace® High-Fidelity DNA Polymerase. Figure S5: mutations on LaG-2/G4 variants generated by different High-Fidelity DNA Polymerases. Figure S6: amino acid mutations on LaG-2 that were generated by conventional gRNA-guided AIDmut1 and AIDmut2. Figure S7: mutations on LaG-2 DNA generated by conventional gRNA-guided AIDmut1. Figure S8: the characteristics of 3×gRNAΔ21-guided base-editors. Table S1: the full sequences of genes, plasmids, and mLaG-2. [file 9823578.f1.zip › Figure S2.jpg]

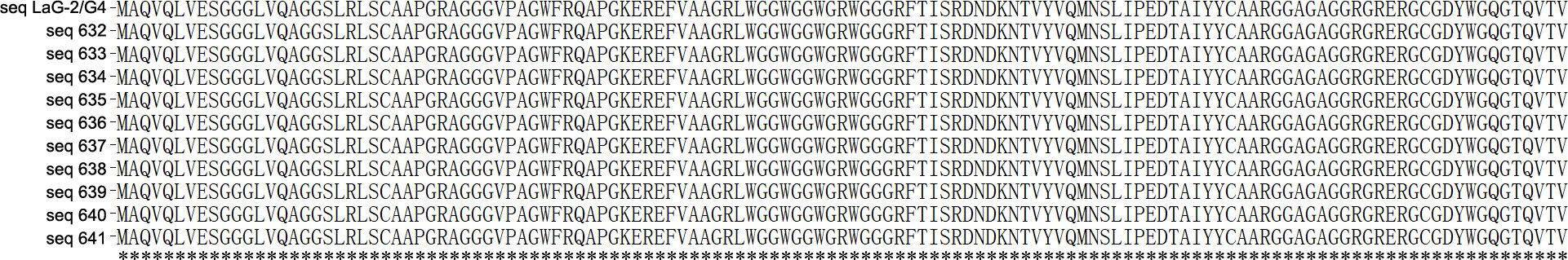

Supplement: Supplementary Materials — Figure S1: G-quadruplex and nucleolin-tethered base-editor-mediated GDP technology. Figure S2: enrichment of the LaG-2/G4 spontaneous mutation at each base in HEK293T cells. Figure S3: enrichment of the LaG-2/G4 spontaneous mutation at each base in Stbl3. Figure S4: mutations on LaG-2/G4 generated by Hieff Canace® High-Fidelity DNA Polymerase. Figure S5: mutations on LaG-2/G4 variants generated by different High-Fidelity DNA Polymerases. Figure S6: amino acid mutations on LaG-2 that were generated by conventional gRNA-guided AIDmut1 and AIDmut2. Figure S7: mutations on LaG-2 DNA generated by conventional gRNA-guided AIDmut1. Figure S8: the characteristics of 3×gRNAΔ21-guided base-editors. Table S1: the full sequences of genes, plasmids, and mLaG-2. [file 9823578.f1.zip › Figure S3.jpg]

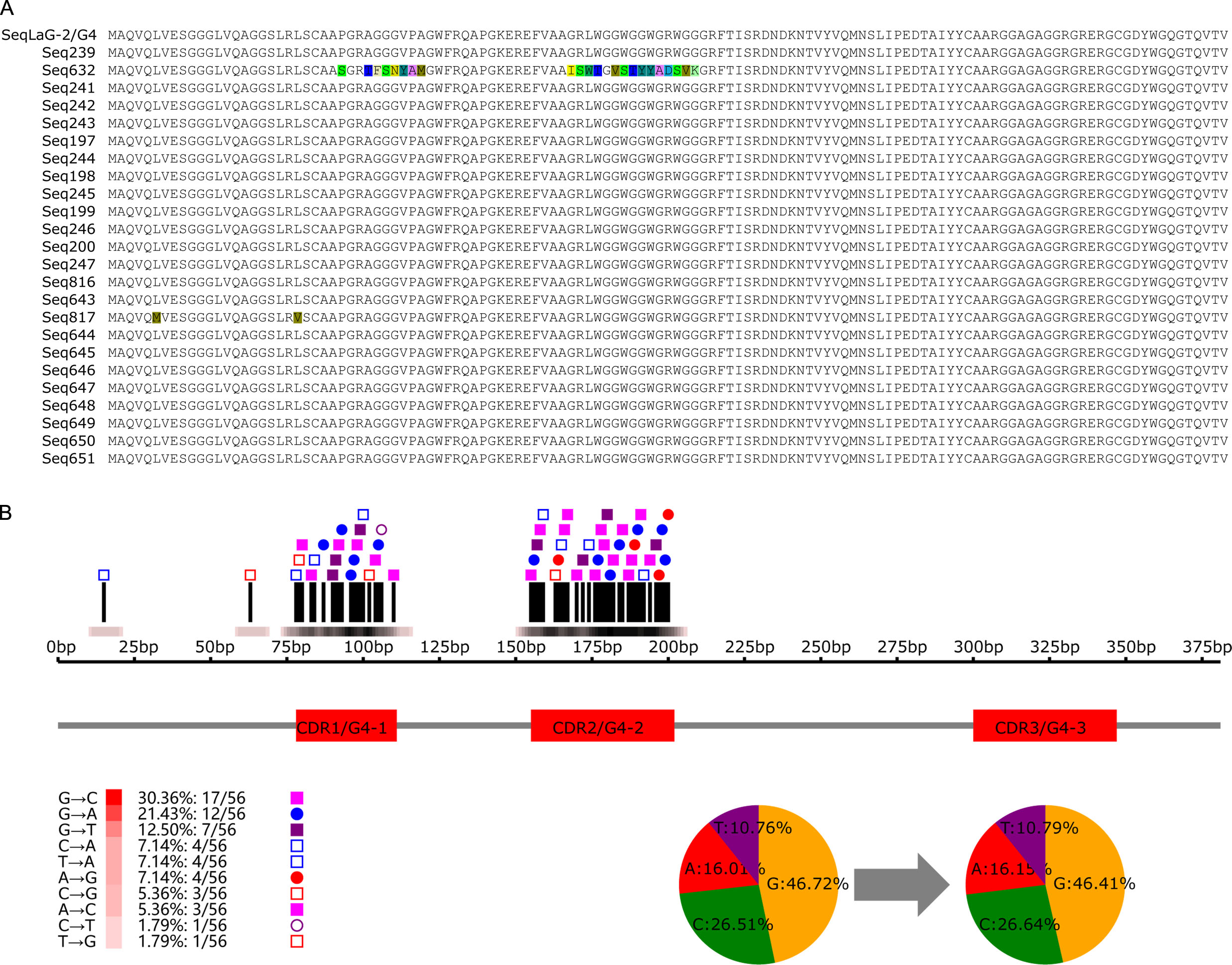

Supplement: Supplementary Materials — Figure S1: G-quadruplex and nucleolin-tethered base-editor-mediated GDP technology. Figure S2: enrichment of the LaG-2/G4 spontaneous mutation at each base in HEK293T cells. Figure S3: enrichment of the LaG-2/G4 spontaneous mutation at each base in Stbl3. Figure S4: mutations on LaG-2/G4 generated by Hieff Canace® High-Fidelity DNA Polymerase. Figure S5: mutations on LaG-2/G4 variants generated by different High-Fidelity DNA Polymerases. Figure S6: amino acid mutations on LaG-2 that were generated by conventional gRNA-guided AIDmut1 and AIDmut2. Figure S7: mutations on LaG-2 DNA generated by conventional gRNA-guided AIDmut1. Figure S8: the characteristics of 3×gRNAΔ21-guided base-editors. Table S1: the full sequences of genes, plasmids, and mLaG-2. [file 9823578.f1.zip › Figure S4.jpg]

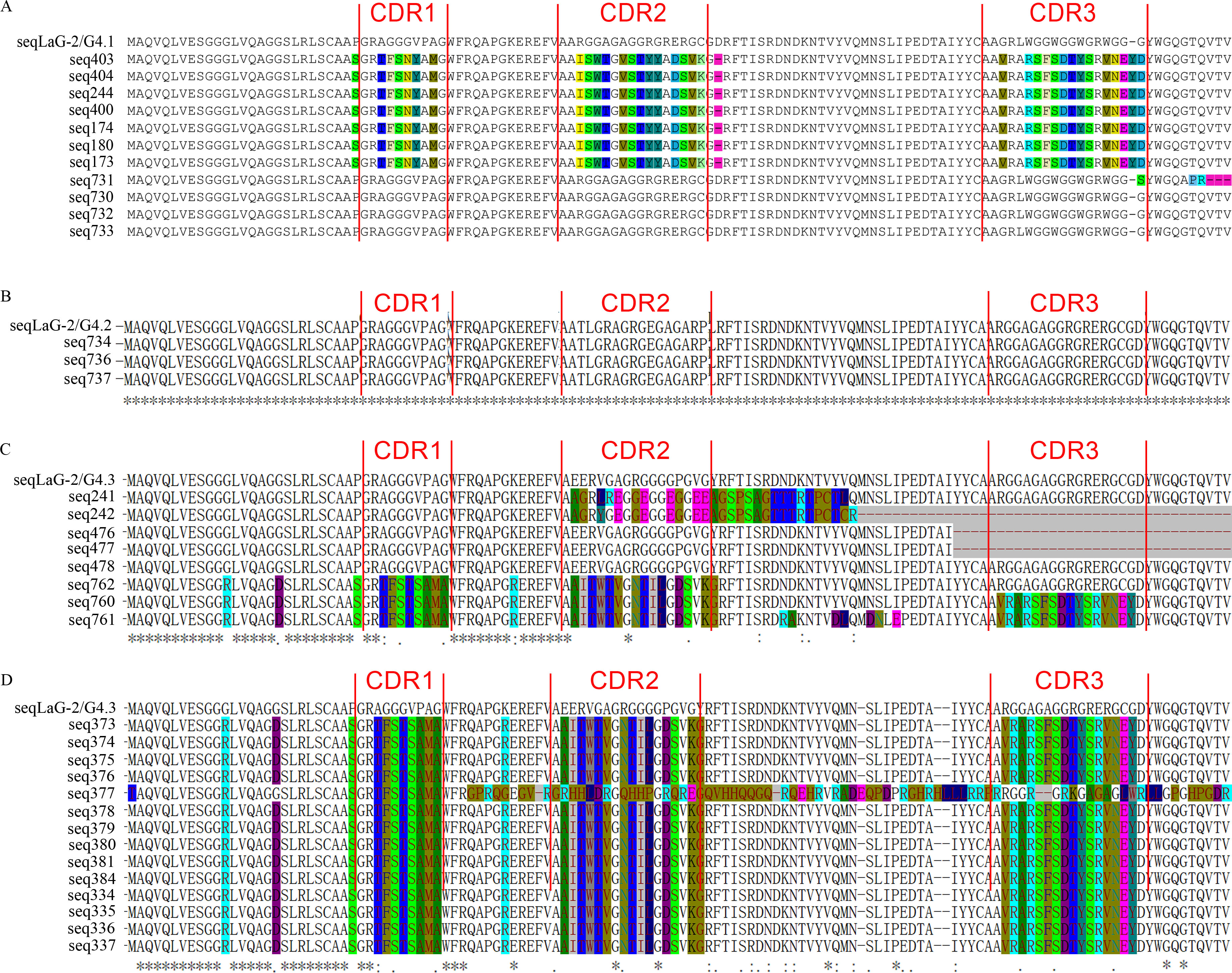

Supplement: Supplementary Materials — Figure S1: G-quadruplex and nucleolin-tethered base-editor-mediated GDP technology. Figure S2: enrichment of the LaG-2/G4 spontaneous mutation at each base in HEK293T cells. Figure S3: enrichment of the LaG-2/G4 spontaneous mutation at each base in Stbl3. Figure S4: mutations on LaG-2/G4 generated by Hieff Canace® High-Fidelity DNA Polymerase. Figure S5: mutations on LaG-2/G4 variants generated by different High-Fidelity DNA Polymerases. Figure S6: amino acid mutations on LaG-2 that were generated by conventional gRNA-guided AIDmut1 and AIDmut2. Figure S7: mutations on LaG-2 DNA generated by conventional gRNA-guided AIDmut1. Figure S8: the characteristics of 3×gRNAΔ21-guided base-editors. Table S1: the full sequences of genes, plasmids, and mLaG-2. [file 9823578.f1.zip › Figure S5.jpg]

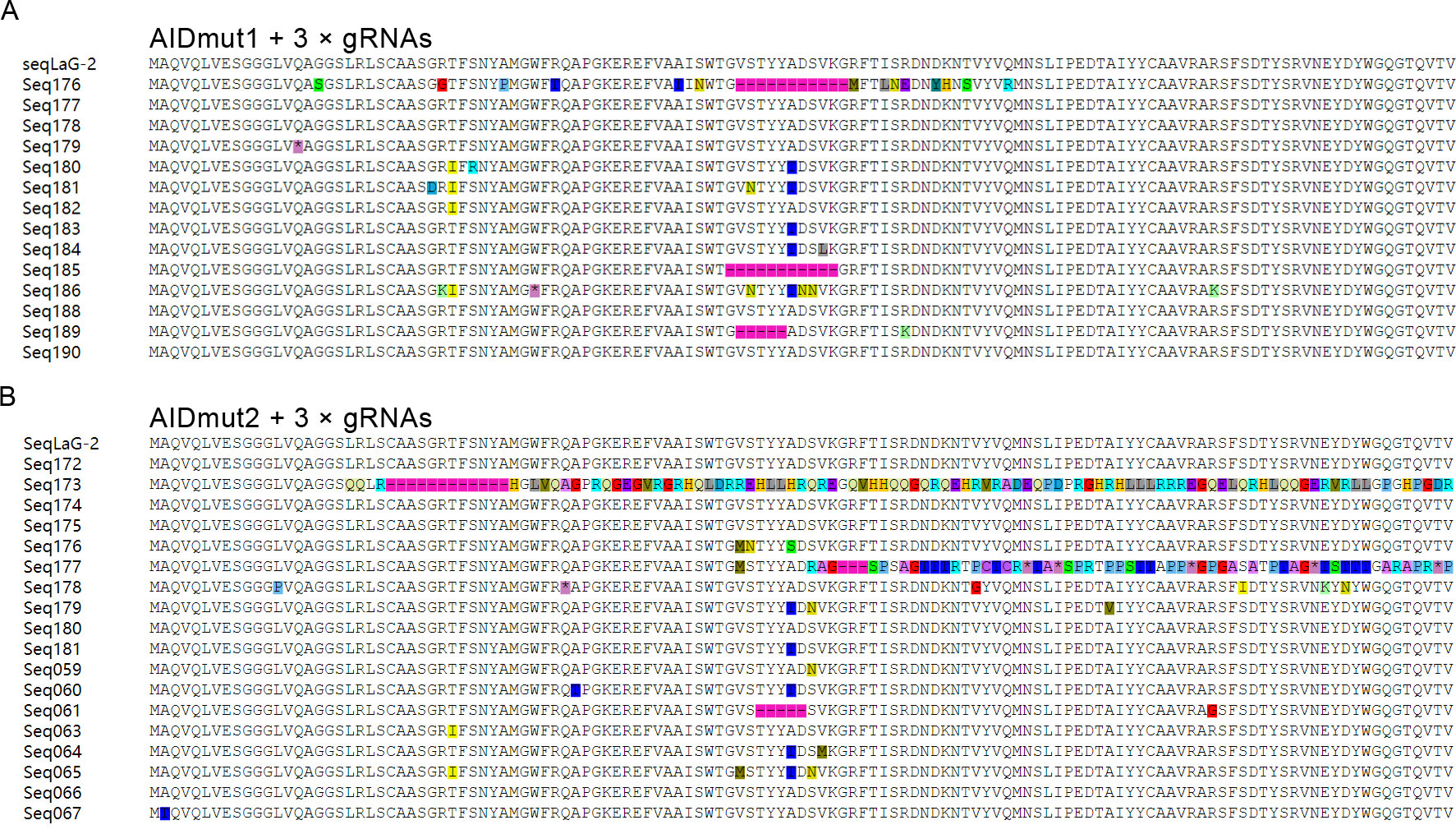

Supplement: Supplementary Materials — Figure S1: G-quadruplex and nucleolin-tethered base-editor-mediated GDP technology. Figure S2: enrichment of the LaG-2/G4 spontaneous mutation at each base in HEK293T cells. Figure S3: enrichment of the LaG-2/G4 spontaneous mutation at each base in Stbl3. Figure S4: mutations on LaG-2/G4 generated by Hieff Canace® High-Fidelity DNA Polymerase. Figure S5: mutations on LaG-2/G4 variants generated by different High-Fidelity DNA Polymerases. Figure S6: amino acid mutations on LaG-2 that were generated by conventional gRNA-guided AIDmut1 and AIDmut2. Figure S7: mutations on LaG-2 DNA generated by conventional gRNA-guided AIDmut1. Figure S8: the characteristics of 3×gRNAΔ21-guided base-editors. Table S1: the full sequences of genes, plasmids, and mLaG-2. [file 9823578.f1.zip › Figure S6.jpg]

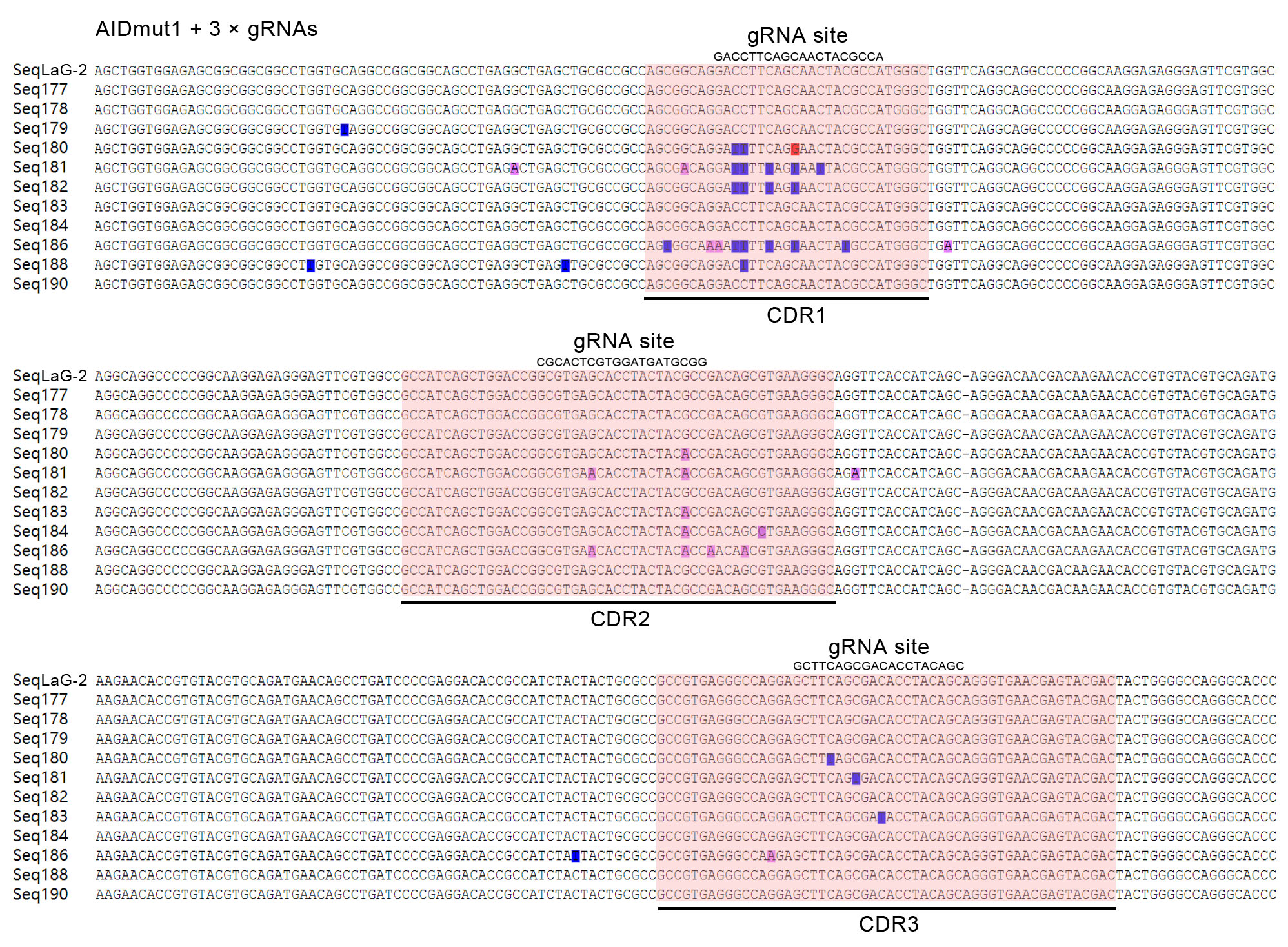

Supplement: Supplementary Materials — Figure S1: G-quadruplex and nucleolin-tethered base-editor-mediated GDP technology. Figure S2: enrichment of the LaG-2/G4 spontaneous mutation at each base in HEK293T cells. Figure S3: enrichment of the LaG-2/G4 spontaneous mutation at each base in Stbl3. Figure S4: mutations on LaG-2/G4 generated by Hieff Canace® High-Fidelity DNA Polymerase. Figure S5: mutations on LaG-2/G4 variants generated by different High-Fidelity DNA Polymerases. Figure S6: amino acid mutations on LaG-2 that were generated by conventional gRNA-guided AIDmut1 and AIDmut2. Figure S7: mutations on LaG-2 DNA generated by conventional gRNA-guided AIDmut1. Figure S8: the characteristics of 3×gRNAΔ21-guided base-editors. Table S1: the full sequences of genes, plasmids, and mLaG-2. [file 9823578.f1.zip › Figure S7.jpg]

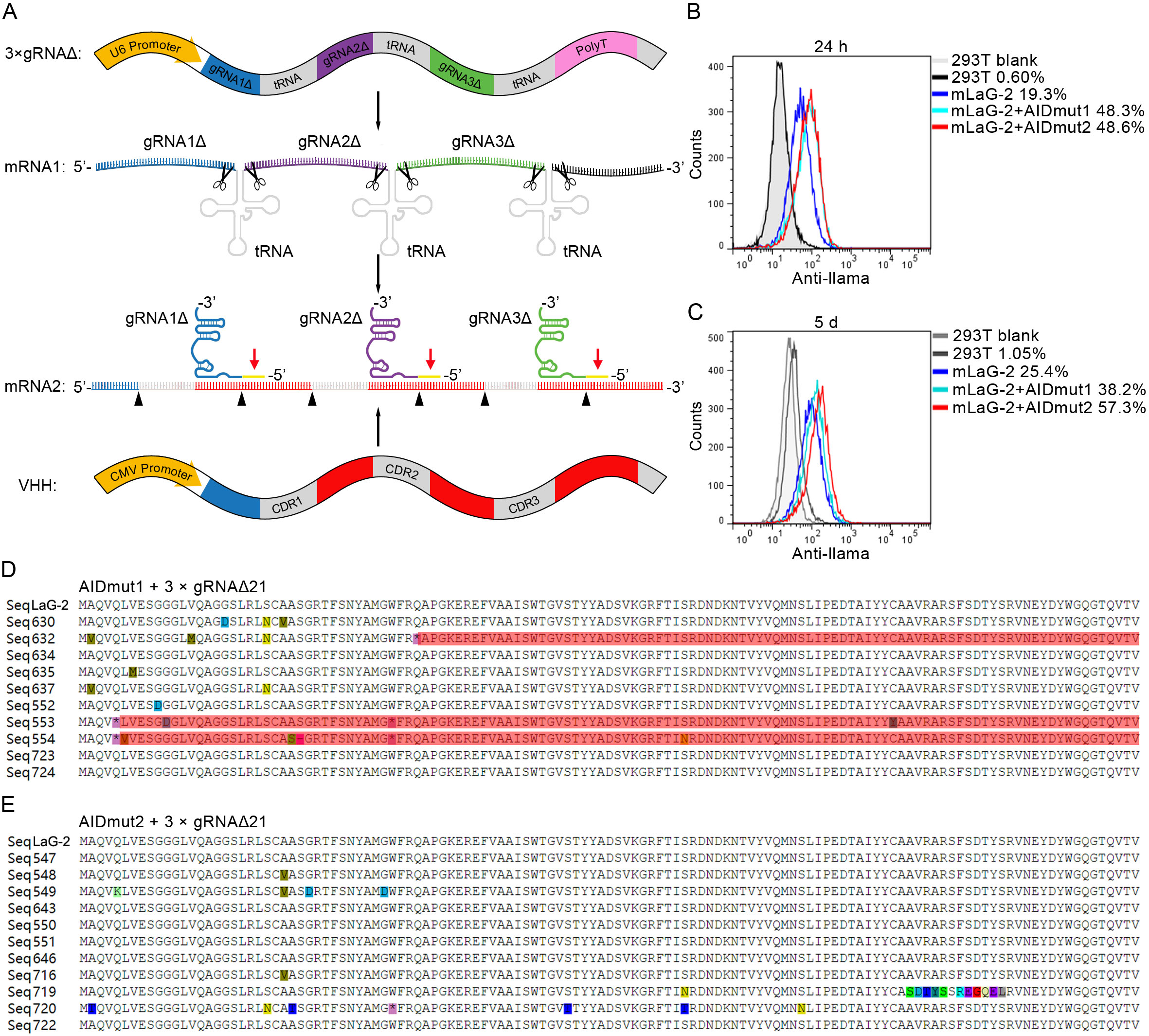

Supplement: Supplementary Materials — Figure S1: G-quadruplex and nucleolin-tethered base-editor-mediated GDP technology. Figure S2: enrichment of the LaG-2/G4 spontaneous mutation at each base in HEK293T cells. Figure S3: enrichment of the LaG-2/G4 spontaneous mutation at each base in Stbl3. Figure S4: mutations on LaG-2/G4 generated by Hieff Canace® High-Fidelity DNA Polymerase. Figure S5: mutations on LaG-2/G4 variants generated by different High-Fidelity DNA Polymerases. Figure S6: amino acid mutations on LaG-2 that were generated by conventional gRNA-guided AIDmut1 and AIDmut2. Figure S7: mutations on LaG-2 DNA generated by conventional gRNA-guided AIDmut1. Figure S8: the characteristics of 3×gRNAΔ21-guided base-editors. Table S1: the full sequences of genes, plasmids, and mLaG-2. [file 9823578.f1.zip › Figure S8.jpg]
